# CWD as a New Health Threat in Europe and the Adequacy and Effectiveness of Instruments of Legal Response from a Comparative Legal Perspective

**DOI:** 10.3390/ani14142027

**Published:** 2024-07-09

**Authors:** Michał Mierkiewicz, Andrzej Dzikowski, Krzysztof Anusz

**Affiliations:** Department of Food Hygiene and Public Health Protection, Institute of Veterinary Medicine, Warsaw University of Life Sciences, 159 Nowoursynowska St., 02-787 Warsaw, Poland; vet.mierkiewicz@gmail.com (M.M.); krzysztof_anusz@sggw.edu.pl (K.A.)

**Keywords:** veterinary law, prion, one health, wild animals

## Abstract

**Simple Summary:**

Unsatisfactory, erroneous, inadequate, or inapplicable laws can lead to negative effects on human and animal health. Such can be the case with the uncontrolled and legally unregulated spread of chronic wasting disease (CWD) with possible zoonotic potential. This disease is spreading in cervids across Europe and other continents. In the context of historical experience with prion diseases, and taking into consideration the “one health” approach, CWD poses a serious challenge. The CWD problem should be properly managed in the European Union before the scale of the disease increases to resemble the situation in North America. The legal comparative analysis conducted in this study indicates that the experience gained by countries that have been struggling with the disease for years should be taken into account when drawing up community legislation.

**Abstract:**

Prions cause infectious and fatal neurodegenerative diseases in mammals. Chronic wasting disease (CWD) affects wild and farmed cervids. The increasing number of cases in Europe, the resistance of prions to external conditions, and the persistence period threaten not only wild cervid populations but also the economy. The possible zoonotic potential of CWD is of growing concern. CWD is a relevant issue as far as the idea of “one health” is concerned, which is a fundamental principle of European veterinary law. Methods of legal text analysis and interpretation are used for this comparative legal study. Research reveals that countries struggling to tackle CWD employ different normative approaches to the problem and use different control and eradication schemes. The results of this study indicate that it is reasonable to issue uniform regulations in the European Union at the common, rather than national, level. The European legislation should creatively draw on the experience of North American countries that have been struggling with the discussed disease for a long time.

## 1. Introduction

Modern public health trends are based on comprehensive global plans, lists of priority pathogens, forecasts, and prevention plans on a supra-local scale. Among factors that could hypothetically cause future pandemics, scientific and social discourse mentions various research hypotheses [1,2]. Diseases caused by prions remain less significant in these efforts than viral or bacterial ones [3].

Transmissible spongiform encephalopathies are caused by prion-altered, infectious protein molecules that replicate without a carrier of genetic information such as DNA or RNA. Prions are formed by converting the host cell’s PrPc prion protein into a modified PrPsc isoform [4,5]. This makes them a potentially major threat to animal and human health [6].

The chronic wasting disease of cervids (hereafter CWD) is the only prion disease found both in wild and farmed animals. It is considered the most contagious prion disease [7,8,9,10]. CWD is constantly spreading to new territories by transmission between wild and farmed cervids [8]. Infectious PrP^CWD^ prions can be found in neuronal tissue and muscles, bodily fluids, and excreted faeces and urine [8,11,12,13,14]. The increasing geographic range of CWD suggests that transmission occurs at least partly via the environment [9,15].

CWD was first observed in farmed deer in 1967 in Colorado, US, and classified as a TSE in the early 1980s [7]. CWD has been reported in 34 continental states in the US and 4 provinces in Canada. It is suspected that the disease may also occur in other parts of Northern America [16]. Currently, the range of the disease extends across several continents [17]. 

In Europe, CWD has been identified in reindeer, elk, and deer in Norway, Finland, and Sweden. The first case in Europe was observed in southern Norway in 2016 in a wild reindeer herd [18]. This led to the launching of an extensive CWD testing program in Norway and several northeastern European Union member states (hereafter EU MSs): Sweden, Finland, Iceland, Estonia, Latvia, Lithuania, and Poland. As a result, CWD cases were detected in Finland, Sweden, and Norway [19,20,21,22,23]. The three-year monitoring program initiated by the European Commission (hereafter EC) [24] was conducted from 2017 to 2020, whereas in Sweden, it was extended until the end of February 2022. 

The epidemic situation is dynamic; existing scientific assumptions regarding the infectious potential and transmission potential of PrP^CWD^ may become outdated at any time [8,25]. 

The purpose of this study is to comparatively analyse the mechanisms of the legal response given to the growing problem of CWD in animal populations in Europe, taking into account the legal experience of North American countries. 

According to the authors, the basic premise for the effective legal reaction is to identify the significance of the disease in the context of public and animal health. Legal response mechanisms must be based on CWD risk management [9,26]. 

The following research hypotheses are put forward: current EU regulations on CWD are insufficient to prevent the spread of the disease in wild cervid populations; legal response mechanisms should be developed for unforeseen crises; the lack of uniform procedures across the EU for monitoring and controlling CWD may lead to variation in the effectiveness of disease prevention and, consequently, further spread.

## 2. Material and Methods

A comparative analysis was conducted on EU legal acts [24,27,28,29,30] and the national statutory acts of EU MSs Sweden [31] and Finland [32,33], as well as on statutory acts of Norway [34,35,36,37]. Norway belongs to the Schengen Area and directly borders the EU by land, which means that local cervid populations can move freely. Laws adopted in Europe have been contrasted with those in the US [38] and Canada [39,40,41,42,43], i.e., the countries where the first cases of CWD were observed and where the longest history of legal response and the most extensive practical experience have been recorded with regard to administrative veterinary control over the discussed disease.

This study is based on methods of analysis and interpretation relevant to legal science methodology. Legal acts were evaluated in terms of their legislative quality and effectiveness as legal instruments, taking into consideration whether they were adequate to the needs of official veterinary inspection and the protection of animal and human health. Nevertheless, the work is interdisciplinary in nature, as it takes into consideration the veterinary and epidemic aspects of the issue in question.

## 3. Results

### 3.1. EU Law

The analysis revealed that the main legal instrument at the community level at the disposal of official EU MSs food control authorities is Regulation 999/2001 [27]. It concerns the prevention, control, and eradication of TSEs and constitutes the EU’s response to the growing risk from bovine spongiform encephalopathy (hereafter BSE). While the act also addresses TSEs other than BSE, it does so only indirectly. The discussed regulation does not indicate any specific or uniform measures to combat CWD. Each EU MS has the autonomy to decide what measures to apply when CWD is detected in cervids [44]. 

In addition, it was found that the regulation is also significant for the functioning of the EU internal market, as it sets out rules for the control and eradication of TSEs in animal production and the marketing of products of animal origin. 

It has been established that the monitoring, control, and elimination of the disease covered by this regulation creates legal implications within the following areas:

- Protection, movement, and transport of wild animals;
- Hygiene of captive animals to be slaughtered, hygiene of meat obtained from them, and hygiene of deer meat obtained through hunting;
- Hygiene and management of animal by-products.

The regulation requires EU MSs to conduct monitoring and inform the EC and other MSs about the emergence of new forms of TSEs [27].

It has been established that although the regulation contains specific provisions applicable to cervids (such as a ban on transferring live cervids from Norway to the EU and hunting decoys containing urine from Norway, Sweden, and Finland), there are no direct orders or prohibitions (e.g., on the use and transportation of meat obtained from cervids in Norway, Sweden, and Finland). In addition, it has been shown that with regard to the import of animal-derived products obtained from deer into the EU from Canada or the US, such legal restrictions are in place. Among objects that can be imported into the EU is only meat from farmed cervids that have been tested for CWD with the use of histopathological, immunohistochemical, and other methods recognised by competent authorities [27,44]. In addition, meat must not come from a herd where CWD has been found or is officially suspected.

The analysis indicates that CWD is also addressed in Regulation 2016/429, shortly referred to as the “Animal Health Law” (hereafter AHL) [28]. This is a general basic act of EU law for the protection of animal health and public health. Although the AHL is a more recent act of law, it has been established that the applicable principle in this case is lex specialis rather than lex posterior. The AHL indicates that provisions on TSEs and other zoonotic diseases that were regulated in previous EU legislation, e.g., Regulation 999/2001, should remain in force to avoid the overlapping of EU law. While the AHL is a broad, general statutory act, Regulation 999/2001 is more specific. Therefore, it has been concluded that the AHL should only be applied to CWD to the extent to which the disease falls beyond the scope of more specific legislation. 

In addition, the AHL also establishes a general guiding interpretive rule for all EU legislation concerning the area under review. Thus, the provisions of the TSE regulation must be interpreted in accordance with the rationes legis (reasons for the law) and the objectives of the AHL, including the “one health” approach. General criteria also apply, such as the AHL classification of animals into either wild or kept categories, regardless of their domestication [28].

Another European statutory act identified and subjected to the current analysis is Directive 2003/99, which deals with the monitoring of zoonoses and zoonotic agents [29]. The ratio legis behind these standards is to ensure adequate epidemiological investigation, collect data necessary for evaluation, and determine the epidemic trends in the EU.

It has been established that implementing Regulation 2019/627 [30] is another significant source of European law. It sets out detailed rules for carrying out official inspections and post-mortem/post-slaughter examinations of cervids intended for human consumption [30]. According to this act, an official veterinarian verifies whether high-risk material has been withdrawn and whether food companies carry out measures to prevent contamination of meat with such material. At the same time, it has been shown that, with regard to cervids, Regulation 999/2001 [27,44] provides no list of tissues or organs to be considered specified risk material, as it does with respect to cattle, sheep, and goats.

Another EU legal act taken under scrutiny is Regulation 2017/1972 [24], introducing a 3-year CWD surveillance program in selected EU MSs. The analysis indicates that this surveillance program was aimed to rule out or confirm the existence of the disease and to determine its prevalence and geographic extent. As a result, CWD was detected for the first time in Finland and Sweden and confirmed in further areas of Norway. 

Research shows that the monitoring of CWD within the EU is based on samples of obex taken for TSE testing [27]. In addition, if possible, samples are taken from the pharyngeal lymph nodes, tonsils, or other lymph nodes of the head. The same rapid tests are used for CWD as in the case of other TSEs: the Bio-Rad TeSeE SAP rapid test with CWD; Idexx HerdChek CWD Antigen Test Kit, EIA; and Idexx HerdChek BSE-Scrapie Antigen Test Kit, EIA [24,27]. In case of inconclusive or positive results, samples are subjected to confirmatory testing using immunohistochemistry or Western blot. The prion protein genotype is then determined for each result [27]. If any EU MS is unable to independently confirm the positive result of a rapid test, the material is sent to a relevant EU reference laboratory [27]. Rapid diagnostic tests have proven effective in identifying various CWD strains, although their use may sometimes lead to false positives [45]. 

### 3.2. National Legislation in Europe

It has been revealed that the abovementioned EU regulations are directly applicable and binding within all EU member states, including Sweden and Finland. Nevertheless, they fail to constitute an exclusive source of law. Each EU MS has the right to establish its national laws and regulations concerning CWD, depending on the given epidemic situation and current requirements. It should be noted that the EU is an international agreement of independent countries that have transferred some of their competences to the community bodies but retain self-determination in most areas. Although the EU has extensive competences in the field of animal health and safety, it is not exclusive—in the absence of a specific legal basis at the European level, each EU MS can (and should) issue national law. This approach allows two neighbouring countries to take similar—yet different—legal measures. 

#### 3.2.1. Sweden and Finland

The rules adopted in Finland and Sweden are based on the implementation of the EU law under scrutiny in the earlier part of this work. The legal methods adopted in the two countries are essentially correspondent and can therefore be considered jointly. It has been concluded that national standards are only secondary in nature, and the burden of producing a legal response to CWD lies on EU law.

Our research revealed that the regulations concerning CWD in Sweden are fundamentally based on Regulation 999/2001. In addition, the disease is subject to reporting in accordance with the Epizootic Diseases Act [31]. The surveillance of suspected cases of CWD is conducted by a competent body of veterinary administration in Sweden, i.e., the Swedish Veterinary Agency. Tests are conducted to further identify sporadic cases of CWD in elk in the Nordic countries, which differs from the typical infectious type of CWD observed in North America and in wild reindeer in Norway [46]. 

Normative regulations on CWD in Finland [32,33] are based on principles of continuous monitoring and mandatory reporting. The classification of animal diseases is regulated by the Animal Diseases Act [32], which stipulates the implementation of European veterinary regulations. Based on this statute, an implementing act was issued [34], classifying CWD under the category of “other animal diseases” that are controllable and notifiable. If a case or a suspicion of CWD is observed, Finnish veterinary administration authorities are obliged to prevent the spread of CWD to the entire country or a specific risk-affected area. Administrative orders and prohibitions for CWD in wildlife have been found to cover movement, isolation, feeding, monitoring, reporting, and disinfection [32,33].

#### 3.2.2. Norway

The analysis showed that the Norwegian Veterinary Institute conducts CWD monitoring based on several legal bases [34,35,36,37]. The analysed Norwegian regulations include numerous implementing acts for the prevention, control, and eradication of TSEs [34], as well as CWD-specific acts [34,35,36,37]. In addition, it has been established that owing to the conclusion of the European Economic Area Agreement (hereafter EEA), Norway takes advantage of EU law [27]. 

The implementing rules [35] concern, for instance, the movement of cervids. It has been found that the movement of reindeer between Norway, Sweden, and Finland is regulated within the studied scope. In addition, there is also an act specifying measures to limit the spread of CWD [36]. This is a detailed piece of legislation dedicated solely to the disease under study. The regulation aims to limit the spread of CWD and supplements the previously mentioned provisions that are broader in scope. It establishes a number of procedures and restrictions on the movement of animals, prohibits the distribution of feed for wild deer, and sets an obligation to notify the local food safety administration of any suspected cases of neurological disorders in cervids. The discussed legal act imposes feeding requirements for reindeer and farmed deer in order to prevent the spread of the disease via the alimentary route and interaction with free-ranging animals. Oversight of enforcement falls to the Norwegian Food Safety Authority. 

In addition, regulations concerning CWD detection zones are present in Norway [37]. They are dedicated solely to the disease in question and cover the following scopes: territorial (valid in specific zones), personal (concerning every citizen), and objective (concerning all cervids). The following activities are prohibited: the distribution of animal feed in open fields; the movement of live deer out of the zones; the movement of lichen and plant material as feed; the movement of dead deer; the display and use of saltlick; and the so-called “guest” grazing of sheep. The ratio legis is to limit the spread of the disease in zones surrounding the locations where an outbreak is detected. 

Moreover, orders have been identified for securing feed on agricultural land and securing elements of dead deer from access by other animals. In addition, these regulations have been found to establish an obligation to clean and disinfect hunting equipment [37]. 

### 3.3. National Legislation in North America

#### 3.3.1. United States of America

CWD regulations in the US, unlike those adopted in Europe, have been found to comprehensively cover the obtaining of meat and animal products from deer. The primary act of legislation is the Code of Federal Regulations [38]. The entity enforcing the regulations is an authorised veterinarian that has received training concerning the disease under review. 

In terms of US legislation, special attention should be given to the CWD Herd Certification Program [38,39]. This program involves a collaborative approach from public authorities and animal owners; it imposes an obligation on the latter to ensure identification, documentation, biosecurity, and notification. In addition, the interstate movement of farmed cervids is restricted, and there is an obligation to maintain health certificates for animals [38].

Moreover, it has been concluded that the normative distinction between CWD-exposed, CWD-positive, and CWD-source animals and herds is a reasonable provision [38] since an open definition could result in a broader meaning, which would be undesirable in this given case. Legal definitions, such as the discussed one, are intended to eliminate different interpretations of phrases and concepts used in legal provisions and their different application in analogous situations.

#### 3.3.2. Canada

The analysis indicated that the Canadian Health of Animals Act [40] is a general law on diseases and toxic substances that can affect animals, zoonoses, and animal protection [41]. It applies to the diseases that can affect not only human health but also the Canadian economy. 

CWD is a notifiable disease that must be reported to the district veterinarian of the Canadian Food Inspection Agency. Mandatory reporting applies to all suspected and confirmed cases [40,42]. 

It has been found that unlike the regulations adopted in Europe but much like those in the US, Canadian regulations comprehensively regulate the obtaining of meat and meat-derived products from cervids [43]. The slaughter of cervids under the veterinary supervision of the Canadian Food Inspection Agency involves the mandatory testing of all slaughtered cervids over 12 months of age [43], and no edible products can be marketed until a negative test result for CWD is obtained. In the event of a positive test result, all parts of the animal must be disposed of using a method that guarantees that prions are destroyed. 

The analysis revealed the significance of the national certification program for CWD-affected breeding herds in Canada [47], even if participation in the program is voluntary and requires a commitment to implementing strict CWD surveillance and biosecurity measures. 

In addition, Canada’s federal government has issued an information booklet on the traditional foods of indigenous peoples, which recommends that no part of animals infected with CWD should be used or consumed by humans. Traditional food obtained through deer hunting has not only nutritional but also significant social, cultural, and spiritual value for Canadian indigenous peoples. The information booklet contains general information about CWD, its signs in animals, the risks it poses, and the means to reduce them. The booklet is a form of social education and popular information, intended to increase public awareness and contribute to detecting the disease in further areas.

## 4. Discussion

Undoubtedly, the emergence of CWD in Europe poses a significant threat to animal and, potentially, human health. Taking into consideration previous TSE outbreaks, a legal response appears necessary. As shown in Section 3, such a response has so far been made in North America and Europe, both at the national and EU level. 

The comparative analysis of the legislation in Finland and Sweden showed that the transparency of data regarding monitoring and the geographical scope of the disease deserves attention. This approach to the problem indicates that CWD is under constant control of local government authorities. The authors assessed the Finnish legal model positively, which is based in principle on EU law, with the simultaneous introduction of one general statutory act and one implementing regulation. Thus, Finnish veterinary administration authorities and deer breeders can transparently find appropriate regulations regarding CWD and monitoring data regarding the geographical scope of the disease. This ensures good information exchange between the legislative and executive authorities and citizens. 

Norwegian legislative activities involving the introduction of orders, prohibitions, and obligations with regard to intracommunity regulations were evaluated positively. It is justifiable and highly reasonable given the long border with Sweden and Norway’s membership in the European Economic Area. Norwegian regulations were found to be relatively clear and understandable, although the existence of several acts of law indicates certain flaws in the quality of the legislation and legislative practice. 

The analysis highlighted the positive effect of the orders, prohibitions, and obligations arising from the aforementioned regulations, as well as the pursuit of compatibility with EU law. At the same time, however, it has been found that not all prohibitions are absolute, and the regulations are casuistic in nature, which constitutes a negative feature. 

This study shows that Norway is trying to regulate the problem under study as much as possible and limit the geographical scope of the disease. Most noteworthy are the feeding requirements for reindeer and farmed deer set to prevent the spread of the disease via the alimentary route and interactions with wild animals. 

It was found that the lack of a vacatio legis period (the period between the announcement of a legal act and its entering into force) in the case of two Norwegian laws, covering measures to limit CWD [36] and zones for its detection [37], was due to the deliberate need to have them implemented immediately. It was also revealed that the legal response in Norway was interventionist in nature and intended to have an immediate or short-term effect in a given place and time. Despite their merits, they are still not thoroughly comprehensive measures that would take into account all possible risks and allow for solving problems likely to arise in the future. 

The results of this study translate to a generally positive evaluation of the US approach to the CWD problem. According to the authors, the CWD Herd Certification Program in particular should provide legislative inspiration for EU lawmakers, given the nature of the AHL, which is a general piece of EU legislation, and the adoption of the “one health” standard. 

Nevertheless, the overabundance of legal definitions in US law deserves a negative assessment. This can make it difficult to determine the relationship between specific items and apply them in practice, as they can limit the flexibility of the law and complicate its enforcement.

The results of this analysis translate to a positive evaluation of the Canadian authorities’ legal approach to the issue in question. Of particular note is the analogy between the Canadian Health of Animals Act and the European AHL. There are no other general implementing regulations in Canada. It is a holistic and clear approach, with no fragmentation between different pieces of legislation. It allows for fighting the disease relatively effectively and limiting the spread of CWD. The existence of preventive measures employed at the slaughter stage is also worth emphasising. 

This study clearly indicates that European legislators, both at the national and European levels, should learn from their US and Canadian counterparts. 

As shown, legislation for the prevention, control, and eradication of TSEs at the EU level is currently based on acts tailored to specific requirements related to BSE but not CWD.

It has been established that even though the EC is undertaking certain measures to control the spread of CWD in Europe, the authors believe they are insufficient and not flexible enough to control the epidemic situation. Although the EC has taken comprehensive measures to monitor and evaluate CWD, their effectiveness, as indicated, depends on a number of factors, including the rate of implementing recommendations and constant adaptation to changing conditions and newly acquired information on CWD. Therefore, as far as this particular disease is concerned, it is vital to ensure a flexible plan of action and clear, general, and adequate legislation. 

In terms of the shortcomings identified, it is of particular note that the current EU law is not tailored to address CWD specifically. Regulation 999/2001 is a relatively old piece of legislation, focusing on managing the risks associated with BSE and scrapie [48]. Unlike in the case of those diseases, however, the control and monitoring of CWD is much more complex due to its occurrence in wild animals, the movement of non-domesticated cervids on a local and global scale, and the considerable duration of prion infectious potential in the environment [10,17,23,25,49,50,51]. In addition, the legislation is primarily concerned with the protection of public health and lacks regulations directly related to animal health and wildlife protection.

The substantial risk connected with CWD in Europe should lead to adopting regulations to minimise the human exposure caused by the introduction of potentially infected cervid meat into the food chain. Such regulations are in place for meat obtained from cattle and small ruminants with regard to TSEs, as well as for pork and game meat with regard to African swine fever, even though the latter disease does not pose a public health risk.

Regulation 999/2001 introduces a series of rules for the trade and import of cattle, sheep, and goats and their meat, as well as the measures undertaken when TSEs are detected in these species. The lack of such specific regulations on CWD and cervids susceptible to the disease has been identified as a major scarcity. 

This flaw in EU law has been found to be due to the novelty of CWD in Europe. It may also stem from practical reasons, such as the fact that the disease occurs mainly in wild animals. On the other hand, it should not be ignored that CWD, which is contagious among cervids, does have potential public health implications. Undoubtedly, due to its etiological agent, CWD is related to other TSEs [52].

If there is suspicion [27] that an animal is infected with any TSE, it must be placed under official movement restriction (e.g., the prohibition of sale to a slaughterhouse or another breeder) until a diagnosis is obtained as a result of clinical and epidemiological tests conducted by relevant public veterinary authorities. Alternatively, it may be slaughtered for laboratory testing under official supervision. This provision is questionable concerning its feasibility with regard to deer in Europe due to being non-domesticated animals.

Following the results of the aforementioned 3-year program [24], Implementing Decision 2016/1918 [53] banned the movement of live cervids into the EU from Norway. The 2016 decision can be evaluated as an immediate measure taken to prevent the geographical spread of the disease; it was a preventive action. In the case under review, each EU MS concerned must implement the EU regulations through internal national acts in order to ensure applicability to entities operating in the country (the decisions of the EC must be implemented, as they generally concern member states; therefore, they must be introduced in each EU MS to become applicable). 

Nevertheless, it has been found that a constitutive regulation prohibiting movement at the community level was not issued until 2022 when a ban was established on the movement of deer from Norway or any other EU MS where CWD was confirmed [54]. 

According to this study, the most desirable legal framework involves issuing a single piece of legislation with the significance of European Regulation to deal specifically with CWD. The legislation should unify, harmonise, and simplify the application of animal law and make it more flexible. Such a legal act should also include a procedural algorithm that would facilitate the work of public veterinary administration across the EU. The goal and ratio legis should be to improve the overall epizootic situation throughout the EU [55]. 

Bringing the CWD problem under the comprehensive regulation on movement, breeding, slaughtering, and sampling will result in improved risk management within the population of wild cervids. Since they can also be farmed, it would be reasonable to introduce a health status assessment for different types of cervid farming facilities. 

In the case of wild animals, it can be assumed that there is a limited likelihood that individuals suspected of clinical CWD will be easily detected or readily identified [56] and then sampled and tested in accordance with current EU standards. Adequate regulation for wildlife is all the more necessary and significant because there are greater control options for domesticated farm animals (e.g., cows) than for non-domesticated animals, even those kept under farm conditions. Sanitary control of deer kept in an enclosed forested territory or reindeer grazed in a semi-free state is very difficult in practice. 

Therefore, the CWD surveillance program should include a larger number of cervids, not just suspected animals. In light of the conducted research, the validity of the European Food Safety Authority‘s recommendation, which took into account the difficulty of diagnosing the disease in wild animals, can be confirmed [23]. 

However, it would be pointless to consider introducing a normative restriction on the movement of wild deer, especially during the mating season. Therefore, the only practical solution seems to be reductive culling to limit contact between individual areas. This was also the current strategy—as a result, the wild reindeer population in the first CWD-affected area in Europe was eliminated by culling [57]. In conjunction with proving that the disease can be transmitted by living in a common environment, reduction culling brings with it indisputable benefits, i.e., removing potential sources of infection from the natural environment and predominating communication between herds.

It is also important to establish regulations for the animal habitat, taking into account the potential of environmental factors for the spread of CWD. The infectious potential of a contaminated environment has been proven for the disease in question [49]. The ability of plants to bind and retain infectious prion proteins, the ability of prions excreted in faeces or urine to bind, and even the ability of plants to take up prions from contaminated soil and transport them to stems and leaves have been demonstrated [58]. Studies on the distribution of PrP^CWD^ in the organisms of susceptible species, soil, and plants indicate that environmental contamination will persist. This is, in the authors’ opinion, a very important aspect, because the previous legislation associated the risk of transmission with the consumption of meat and animal products. The latest scientific research shows, however, another potential risk vector that should be taken into account in the context of risk analysis and assessment, e.g., collecting deer antlers [58,59,60,61].

Our research indicates that monitoring and imposing orders and prohibitions on live animals and their products are currently the only way to prevent the spread of the disease in Europe beyond the Scandinavian Peninsula. In terms of monitoring the disease, both from the normative and veterinary perspectives, it is worth relying on the experience concerning the spread of the disease in North America. 

Once CWD develops in an area, the risk of infection may persist in the environment for a long time, and the territorial extent of the disease is likely to continue expanding [12,15,16,58,59,60,61]. The uncertain but potentially significant risk related to CWD transmission to humans will increase if the disease spreads territorially and the number of infected animals increases [50,62]. EU law should tackle CWD by using the “one health” approach [9,51,63], before the same health and economic consequences as those in the US and Canada are observed in Europe. With this in mind, special attention should also be given to the epidemic situation along the EU eastern border.

## 5. Conclusions

This research revealed and identified risks and deficiencies within EU law. First and foremost, there is a lack of a community-level legal framework for a unified approach and the introduction of official CWD control measures that can be applied when the disease is detected in countries other than those so far affected by CWD. In addition, it was found that EU regulations focus on BSE and its specific characteristics, which are not comparable to CWD. Moreover, legal system differences between EU MSs create the risk that national laws will be incompatible and that the measures to be introduced may prove overly flexible, overly restrictive, or simply too late.

Therefore, the uniform normative regulation of the CWD problem in the EU is advocated. These legal tools should be produced at the common level in advance. Preventive action and anticipation of certain risks can limit the consequences in case of occurrence. As the Latin proverb states “*Si vis pacem, para bellum*” (If you want peace, prepare for war), it is necessary to set up action plans that would prevent the spread and possible unwitting introduction of PrP^CWD^ into the human food chain. In all EU countries, this could be regulated with a screening order, as well as orders and prohibitions on the movement of cervid animals and meat from CWD-affected areas. The repercussions of failing to implement EU-wide biosecurity measures are currently impossible to assess.

## Data Availability

The original contributions presented in the study are included in the article, further inquiries can be directed to the corresponding author.
